# Emerging Roles for Ciz1 in Cell Cycle Regulation and as a Driver of Tumorigenesis

**DOI:** 10.3390/biom7010001

**Published:** 2016-12-27

**Authors:** Tekle Pauzaite, Urvi Thacker, James Tollitt, Nikki A. Copeland

**Affiliations:** Faculty of Health and Medicine, Biomedical and Life Sciences, University of Lancaster, Lancaster LA1 4YQ, UK; t.pauzaite@lancaster.ac.uk (T.P.); u.thacker@lancaster.ac.uk (U.T.); j.tollitt@lancaster.ac.uk (J.T.)

**Keywords:** CDK, cell cycle, cancer, DNA replication stress, genome stability

## Abstract

Precise duplication of the genome is a prerequisite for the health and longevity of multicellular organisms. The temporal regulation of origin specification, replication licensing, and firing at replication origins is mediated by the cyclin-dependent kinases. Here the role of Cip1 interacting Zinc finger protein 1 (Ciz1) in regulation of cell cycle progression is discussed. Ciz1 contributes to regulation of the G1/S transition in mammalian cells. Ciz1 contacts the pre-replication complex (pre-RC) through cell division cycle 6 (Cdc6) interactions and aids localization of cyclin A- cyclin-dependent kinase 2 (CDK2) activity to chromatin and the nuclear matrix during initiation of DNA replication. We discuss evidence that Ciz1 serves as a kinase sensor that regulates both initiation of DNA replication and prevention of re-replication. Finally, the emerging role for Ciz1 in cancer biology is discussed. Ciz1 is overexpressed in common tumors and tumor growth is dependent on Ciz1 expression, suggesting that Ciz1 is a driver of tumor growth. We present evidence that Ciz1 may contribute to deregulation of the cell cycle due to its ability to alter the CDK activity thresholds that are permissive for initiation of DNA replication. We propose that Ciz1 may contribute to oncogenesis by induction of DNA replication stress and that Ciz1 may be a multifaceted target in cancer therapy.

## 1. Introduction

Regulation of DNA replication is required to ensure that the genome is precisely duplicated prior to segregation into daughter cells. Faithful duplication of the genome is central to the long-term health of an organism. This process is regulated at multiple levels to ensure near-perfect chromosome duplication with error rates at less than 1 per billion bases copied [1]. This level of precision requires highly orchestrated and stratified mechanisms to ensure that DNA replication occurs once and only once per cell cycle. DNA replication initiates from multiple sites that are marked by the pre-replication complex (pre-RC, (origin replication complex (ORC), cell division cycle 6 (Cdc6), chromatin licensing and DNA replication factor 1 (Cdt1), mini-chromosome maintenance 2-7, (Mcm2-7)) [2,3,4]. This complex process is conserved in eukaryotes and putative replication origins are activated by the concerted activities of cyclin E- cyclin-dependent kinase 2 (CDK2) and Dbf4-dependent kinase (DDK) [5,6,7] through activation of the Cdc45, Mcm2-7 and the GINS complex consisting of Sld5, Psf1, Psf2, and Psf3 (‘Go Ichi Ni San’) that are collectively referred to as the CMG complex [6,8,9]. Activation of the CMG complex facilitates loading of processivity factor - proliferating cell nuclear antigen (PCNA) and DNA polymerases to complete the replisome [6,9,10]. This process is conserved from yeast to man, but in vertebrates there are additional factors that contribute to efficient and timely DNA replication. This review will describe the function of Ciz1 in maintenance of genome integrity by regulation of the G1/S transition and evaluate its role in cancer biology.

## 2. Temporal Regulation of Replication Complex Assembly

Origin specification, replication complex assembly and activation are highly conserved processes in eukaryotes. To ensure timely replication of large genomes, eukaryotes use multiple replication origins that are spaced approximately 50–100 kb throughout the genome [11,12,13]. These sequences are specified by the autonomous replicating sequence (ARS) in yeast, but in higher eukaryotes putative replication origins do not have defined sequences [14]. In higher eukaryotes, origin specification begins with ORC binding to AT-rich DNA sequences with permissive epigenetic markers, which are regulated by recruitment of chromatin remodeling factors [13,15]. ORC binding preferentially occurs at open chromatin with Histone 3 (H3) acetylation and Histone 3 Lysine 4 (H3K4) methylation that correlate an increased number of putative replication origins in euchromatin relative to heterochromatin. This bias towards “open” chromatin correlates with increased frequency of fragile sites in heterochromatin due to a paucity of putative origins [4,15].

**Figure 1:**
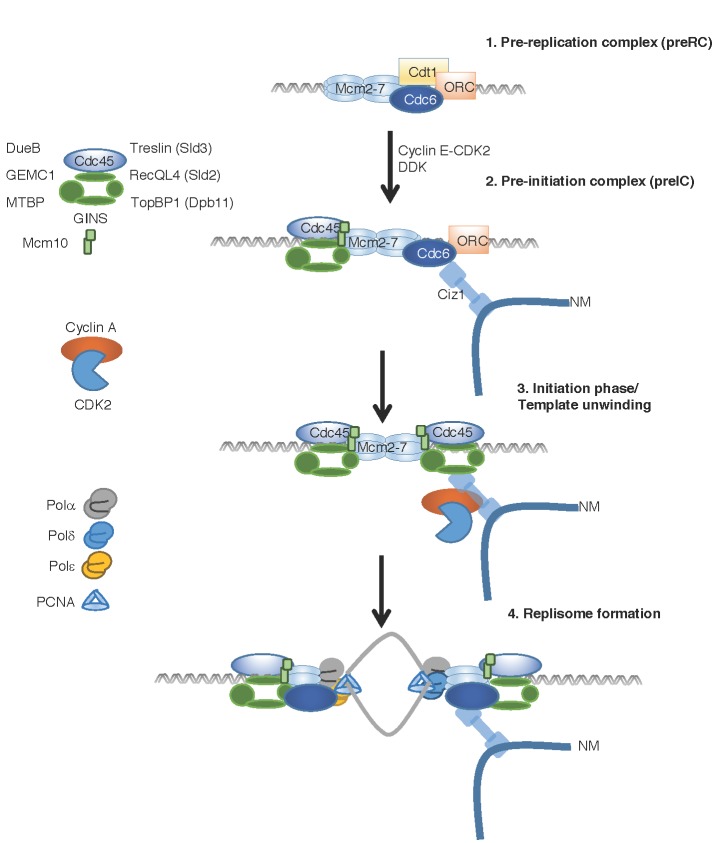
Replication complex assembly from origin specification to replisome formation. Replication origin specification by origin replicating complex (ORC) binding, enables recruitment of cell division cycle 6 (Cdc6), chromatin licensing and DNA replication factor 1 (Cdt1) and mini-chromosome maintenance 2-7 (Mcm2-7) in an ATP-dependent manner [20,21,22] completing formation of pre-replication complex (pre-RC). Completion of the pre-RC at the origin is referred to as replication licensing and does not require cyclin-dependent kinase (CDK) activity. Ciz1 binds to the nuclear matrix (NM) where it associates with Cdc6, mediating chromatin/nuclear matrix contacts [23]. The ORC-Cdc6 complex is separated from the double Mcm2-7 hexamer structure before pre-initiation complex (pre-IC) formation [21,24]. Cyclin E-CDK2 and Dbf4-dependent kinase (DDK) cooperate to facilitate loading of the Cdc45-Mcm2-7-GINS (‘Go Ichi Ni San’) (CMG complex) onto chromatin [7]. Pre-IC assembly requires DDK- and CDK-mediated recruitment and activation of the synthetic lethal with Dpb11-1 2 and 3 - DNA polymerase B-associated protein 11 (Sld2/3-Dpb11) complex to efficiently load the active helicase, CMG. Loading of the CMG complex in vertebrates is also promoted by additional factors including DNA unwinding element B (DUE-B), Geminin coiled-coil domain-containing protein 1 (GEMC1) and Mouse double minute 2 (MDM2) binding protein (MTBP) [25,26,27,28,29,30]. Ciz1 is a nuclear matrix-associated DNA replication initiation factor, which recruits cyclin E-CDK2 and Cyclin A–CDK2 [31] to facilitate initiation of DNA replication [23]. The final step in replisome formation is recruitment of polymerases and accessory factors that enable precise, processive DNA replication. Other abbreviations: TopBP1: DNA Topoisomerase II binding protein; DDK: Dbf4 dependent kinase; PCNA: proliferating cell nuclear antigen; Polα: DNA polymerase alpha; Polδ: DNA polymerase delta; Pol ε: DNA polymerase epsilon.

ORC serves as a platform for further recruitment of factors to facilitate DNA replication (Figure 1). The process of pre-RC assembly begins by recognition of ORC leading to loading of Mcm2-7 complex at putative replication origins by Cdc6 and Cdt1 [16,17]. Mcm2-7 loading is mediated by sequential rounds of binding and release of Cdc6 and Cdt1 at individual origins. Each round loads a single Mcm2-7 complex, resulting in two Mcm2-7 complexes loaded in opposing orientations [3,18]. There is an excess of Mcm2-7 complex at each replication origin and replication timing is influenced by the efficiency of Mcm2-7 complex supra-loading [19]. The resulting pre-replicative complex (pre-RC) completes replication licensing at the associated origin. The licensed origin remains inactive until S phase and activation requires loading of additional binding partners and phosphorylation by CDK and DDK [5,6].

Formation of pre-RC serves as a base for recruitment of initiation factors leading to the formation of pre-initiation complex (pre-IC). This complex consists of Cdc45, Mcm2-7 and GINS that form the active helicase which associates with the replisome during S phase [32]. The mechanism for pre-IC formation is conserved in eukaryotes and requires the combined activity of CDK and DDK for formation and activation. Dbf4-dependent kinase activates the CMG complex [5] and CDK-mediated phosphorylation of Sld2 and Sld3 leads to Dpb11 binding that promotes CMG loading and activation of Mcm helicase activity [33,34,35,36,37]. Sld3/Treslin coordinates Cdc45 recruitment to Mcm2-7 with DDK phosphorylation of Mcm2 during S phase [38]. In addition, Mcm10 interacts with TopBP1^Rad4^ at origins of DNA replication [39] and aids with the recruitment of Cdc45 after CDK and DDK activation [40]. The temporal loading of Mcm10 occurs via direct interactions with Mcm2-7 resulting in a low affinity complex. This complex then serves to facilitate associations with the CMG complex resulting in a higher affinity complex [41]. Mcm10 coordinates the assembly of the helicase with phosphorylation of the helicase, a critical mechanism to ensure that Mcm2-7 double hexamer complexes are assembled and activated in a coordinated manner [7,41].

These events are conserved in higher eukaryotes which also combine developmental programs with cell cycle control. Consequently, additional replication factors have evolved, which have little homology with “core” conserved replication proteins. In *Xenopus* and human cells, the Sld3 homologue Treslin binds to TopBP1 (Dpb11) after CDK-mediated phosphorylation [25,26,27]. In addition, higher eukaryotes have evolved parallel degenerate mechanisms that facilitate pre-IC formation, which are absent in lower eukaryotes, mediated by DNA unwinding element B (DUE-B), Geminin coiled-coil domain-containing protein 1 (GEMC1) and Mouse double minute 2 (MDM2) binding protein (MTBP). Downstream recruitment of MTBP is required for efficient loading of Cdc45/Mcm/GINS and initiation of DNA replication [28]. TopBP1 binds to GEMC1, and this complex recruits Cdc45 to promote DNA replication in *Xenopus* [30]. In a parallel pathway, DUE-B directs efficient recruitment of CMG complex to activated replication origins [29].

The final step is the CDK-mediated recruitment of polymerases and accessory factors, which complete replisome assembly. In mouse fibroblasts re-entering the cell cycle, this step requires Ciz1 for efficient localization of cyclin A-CDK2 to chromatin for initiation of DNA replication [42]. The process of DNA replication requires three replicative polymerases—Pol α, Pol δ, and Pol ε—which associate with the processivity factor proliferating cell nuclear antigen (PCNA) [34,43]. Pol α produces RNA primers on both leading and lagging strands that enable processive DNA replication. DNA polymerases α and ε perform leading-strand synthesis and Pol α and Pol δ perform lagging-strand synthesis with high nucleotide selectivity and efficient proofreading [44]. Ctf4 is required for sister chromatid adhesion and for the stable assembly of the CMG complex and polymerases in the replisome [32,45]. The leading strand Pol ε is positioned ahead of CMG helicase, whereas Ctf4 and the lagging-strand polymerases Pol α/δ are behind the helicase [9,32,44,46]. Together these complexes mediate high fidelity duplication of the genome.

## 3. Discovery of Ciz1 and Its Role in Cell Cycle Regulation

Ciz1 was discovered in a *S. cerevisiae* yeast two-hybrid screen that identified cyclin E-p21 binding partners, although Ciz1 could interact with p21 directly [47]. Ciz1 appears to be unique to vertebrates; it is conserved in mammals, with partial conservation in birds and fish. Ciz1 is a non-essential gene in mice, with Ciz1 null mice showing no severe developmental defects [48]. However, Ciz1 interacts with several proteins that contribute to regulation of cellular proliferation, including transcriptional regulators, cell cycle regulators including cyclin E, cyclin A, CDK2, p21 and proteins that are not directly related to DNA replication (Appendix A Table A1 and references therein). The only functional interactions sites within Ciz1 that have been identified thus far are the conserved cyclin-binding motifs that mediate direct interactions with cyclin A2 and cyclin E [42]. Mutation of the cyclin-binding motifs demonstrated that Ciz1 interactions with cyclin E and cyclin A-CDK2 are essential for its DNA replication function, as mutations within Ciz1 that prevent cyclin binding are inactive in cell-free DNA replication assays [42]. In addition, Ciz1 contributes to cell cycle regulation, spermatogenesis and possibly cancer biology through direct interactions with cyclin A1/A2 that correlate with Ciz1 function in DNA replication and DNA repair respectively [31,42].

The domain structure of Ciz1 is consistent with the role in spatial coordination of DNA replication complex assembly: The *C*-terminus contains the nuclear matrix-binding domains [49] and the *N*-terminus contains the binding sites for pre-RC protein Cdc6 and cyclin A-CDK2 [23,42]. This structural architecture could mediate binding interactions of the pre-RC via Cdc6, cyclin A-CDK2 and the nuclear matrix.

The nuclear matrix contributes to nuclear compartmentalization of factors that contribute to temporal and spatial regulation of transcription and DNA replication [49,50]. The nuclear matrix is an insoluble structure that associates with cell cycle regulators and enzymes required for DNA synthesis [51,52]. Replisomes are associated with the nuclear matrix and retain activity after nuclear fractionation [51,52]. Ciz1 is localized to the nuclear matrix by matrin-like zinc finger domains in the *C*-terminus of Ciz1 [47,49], where it colocalizes with PCNA in S-phase cells, contributing to the G1/S transition and increased cellular proliferation [49,53]. The localization of Ciz1 proximal to active replication factories in G1 phase and colocalization with PCNA during S phase [49,53] suggests that Ciz1 contributes to localization and recruitment of cell cycle regulators for efficient initiation of DNA replication at the nuclear matrix.

## 4. Ciz1 Is a CDK Sensor That Promotes Initiation of DNA Replication and Prevention of Re-Replication

Ciz1 interacts with multiple DNA replication proteins and cell cycle regulators including p21. Overexpression of p21 promotes Ciz1-p21 nuclear exit and sequestration of p21 by Ciz1 was suggested to be a potential mechanism to increase CDK2 activity in G1 phase [46]. However, the sequestration of p21 by Ciz1 is not essential for Ciz1 DNA replication function, as p21 null murine embryonic fibroblasts (MEF) cells are more proliferative after Ciz1 overexpression [53]. In contrast, Ciz1 DNA replication function is dependent on binding to cyclin E and cyclin A at conserved cyclin binding motifs in the DNA replication domain [42]. Both cyclins and CDK2 interact with Ciz1 at defined sites, forming a bipartite interaction site consistent with many cyclin substrates [54]. Ciz1 facilitates cyclin A-CDK2 chromatin localization in late G1 phase via direct interactions at a conserved cyclin-binding motif and mutation of this cyclin-binding site (Cy motif) prevents cyclin binding and blocks Ciz1 DNA replication function [42]. Both short interfering RNA (siRNA)-mediated depletion of Ciz1 and Cy-motif mutation prevent recruitment of cyclin A to chromatin at the G1/S transition [42]. Therefore, Ciz1 promotes the correct sub-nuclear localization of cyclin A-CDK2 at the G1/S transition for efficient initiation of DNA replication [23,42].

Ciz1 function is directly regulated by cyclin A-CDK2 interactions and CDK-mediated phosphorylation. Ciz1 accumulates during G1 phase as cyclin A expression increases at the G1/S transition [23]. Although Ciz1 is an in vitro cyclin E-CDK2, cyclin A-CDK2 and DDK substrate, the in vivo relevance for potential regulatory phosphorylation sites remain to be fully addressed. There are 16 putative CDK phosphorylation sites in murine Ciz1. Only those within the DNA replication domain have been studied, three of which (T144, T192, T293) down-regulate Ciz1 DNA replication function. Importantly, both CDK-mediated phosphorylation of Ciz1 and phosphomimetic mutation of these three sites prevent cyclin A-CDK2 binding [23]. These observations lead to the proposed model where Ciz1 serves as a kinase sensor recruiting cyclin A-CDK2 to putative replication origins via interactions with Cdc6. At low kinase levels, Ciz1 interacts directly with cyclin A-CDK2 to facilitate recruitment to chromatin that is required to promote initiation of DNA replication [42]. However, at higher CDK levels, Ciz1 is phosphorylated on multiple sites and in this hyperphosphorylated form, Ciz1 can no longer associate with cyclin A-CDK2. Hyperphosphorylation of Ciz1 prevents localization of cyclin A-CDK2 to the nuclear matrix, preventing replisome assembly and initiation of DNA replication [23]. Therefore, Ciz1 recruits cyclin A-CDK2 activity to chromatin at the right time and place to ensure efficient initiation of DNA replication.

In addition, Ciz1 contributes to mechanisms that prevent re-replication in a high CDK context through prevention of cyclin A-CDK2 recruitment to chromatin and the nuclear matrix. Ciz1 functions as a CDK sensor that integrates CDK activity and initiation of DNA replication at defined sites [23]. Therefore, Ciz1 functions as a rheostat-like regulatory switch, whereby Ciz1 can respond to an increasing gradient of CDK activity through phosphorylation at multiple sites during the G1/S transition to maintain genome stability.

## 5. Replication Licensing and Prevention of Re-Replication Is Regulated by Cyclin-CDK Complexes

**Figure 2:**
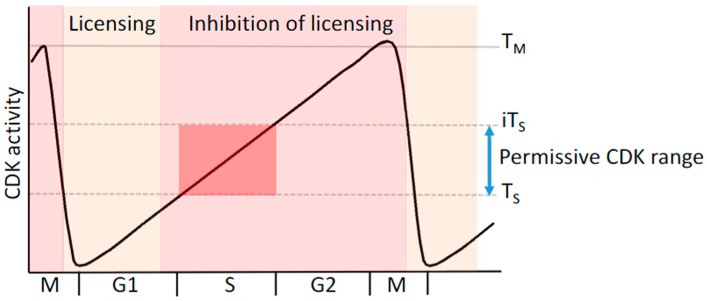
Quantitative model of CDK activity define cell cycle transitions. Cyclin-CDK activity oscillates through the cell cycle defining key transition thresholds, threshold at S phase (T_S_) and mitosis (T_M_) that mark the G1/S transition and G2/M transition respectively. CDK activity also regulates temporal regulation of the replication licensing phase (cream) from the replication initiation phase (light red) where licensing is actively inhibited by high CDK activity. The permissive activity range for CDK activity for initiation of DNA replication (red) and at higher concentrations initiation is blocked until the subsequent cell cycle. The inhibitory concentration of CDK activity is denoted as iT_S_.

Cyclin-dependent kinases are the key regulators of the cell cycle and their oscillating activity contributes to separation of replication licensing from replication origin firing, thereby restricting replication of their genome once per cell cycle [55,56]. In cycling cells, pre-RC assembly begins during late mitosis [57] as the CDK activity drops due to anaphase promoting complex/cyclosome (APC/C) activation, cyclin degradation and phosphatase activation. In quiescent cells re-entering the cell cycle, pre-RC formation is facilitated by the activity of CDK and DDK during G1 phase [35,58,59,60]. In both quiescent and cycling cells, separation of the replication licensing phase from the activation of replication origins is mediated by rising CDK activity (Figure 2). This quantitative model of CDK function defines thresholds that demarcate boundaries within, and between, stages of the cell cycle [61,62,63].

CDK activity temporally regulates replication origin specification and pre-RC assembly, restricting these events to early G1 phase when CDK activity is low. As CDK activity rises during mid- to late-G1 phase this process is inhibited. Inhibition of pre-RC formation is mediated by steric exclusion of pre-RC assembly by direct cyclin binding [64,65,66] or CDK phosphorylation of the pre-RC components Orc2/6, Cdc6 and Cdt1 [65,66,67]. Phosphorylation of Orc2/6, Cdc6 and Cdt1 results in relocalization to the cytosol or ubiquitin proteasome system-mediated destruction that prevents inappropriate re-licensing at high CDK activity [66,68,69,70].

In addition, if CDK activity is too high, phosphorylation of DNA pol α and Ciz1 prevent DNA replication at the G1/S transition. Cyclin A-CDK2 activity is required to both activate DNA pol α during the initiation phase of DNA replication and inhibit its function at high, non-permissive CDK activity [71,72]. This bifunctional response to increasing CDK activity is also seen for Ciz1, which promotes DNA replication in complex with cyclin A-CDK2 at low kinase levels. However, at high CDK activity, Ciz1 is phosphorylated at multiple sites that prevent interaction with cyclin A-CDK2, which is required to promote initiation [23,42]. Therefore, CDK activity contributes to the regulation of origin assembly, activation of replication origins and prevention of re-replication at non-permissive concentrations in late stages of the cell cycle.

## 6. Ciz1 Is a Driver of Tumor Growth

Ciz1 is associated with tumor growth in small cell (SCLC) and non-small cell lung carcinoma (NSCLC) [73], colorectal [74,75], breast [76,77], prostate [78], hepatocellular carcinoma [79] and gall bladder cancer [80]. In each case, there is a cancer-specific alteration resulting in increased Ciz1 protein levels or alternative splicing of *Ciz1* transcript. Deregulation of Ciz1 transcript and protein levels are required for proliferation, invasiveness and anchorage-independent growth in cancer cell lines in vitro (Table 1) [73,74,77,78,79,80,81]. Importantly, targeting cancer-specific splice variations or depletion of Ciz1 by siRNA reduced tumor growth in xenograft models, identifying a potentially selective therapeutic avenue to reduce tumor proliferation [73,74,77,78,79,80,81]. 

In addition, in Ciz1 null mouse models, ablation of Ciz1 predisposes mice to viral transformation, suggesting that it may have a tumor suppressor function [48]. There are currently no other reports that demonstrate that Ciz1 is commonly inactivated in tumors, whereas Ciz1 overexpression has been found to maintain tumor growth in several studies. Taken together, evidence supports the view that some cancers display gene addiction for Ciz1, or that Ciz1 may be a driver of tumor growth in common tumor types. These observations suggest that Ciz1 may contribute to the proliferation and adaption of cells at different stages of tumorigenesis. The dependence on continued expression of Ciz1 for tumor growth and other cancer-specific characteristics suggest that Ciz1 may be a multifaceted target in cancer therapy.

## 7. Ciz1-Mediated Transcriptional Regulation of Tumorigenesis

Ciz1 may promote tumor growth by contributing to deregulation of oncogenic transcription in breast cancer, colorectal carcinoma and gall bladder cancer. In each case, Ciz1 increased tumorigenicity through activation of oncogenic transcription programs. In estrogen-sensitive breast cancer cell lines, *Ciz1* is an estrogen receptor (ER)-dependent transcript that contributes to hypersensitization to estrogen-signaling pathways [77]. Estrogen sensitivity is exacerbated by a positive feedback mechanism, as Ciz1 protein sensitizes cells to estrogen and promotes its own expression via ER-mediated transactivation [77]. In estrogen-sensitive breast cancer cell lines, proliferation was dependent upon overexpression of Ciz1 for enhanced tumor growth in xenograft models and Ciz1-dependent tumor growth was blocked by siRNA-mediated depletion [77].

Ciz1 is a potential prognostic marker of colorectal carcinoma (CRC). Examination of primary CRC tumors and paired patient-derived normal tissues demonstrated that high Ciz1 transcript levels correlate with poor patient survival [75]. Ciz1 is overexpressed in aggressive CRC tumors, which are Ciz1-dependent for tumor proliferation [74]. Ciz1 was found to directly associate with the oncogenic transcription factor Yes associated protein 1 (YAP) and promote higher order interactions with Tafazzin (TAZ) and TEA Domain Transcription Factor 1 (TEAD) that are commonly activated in cancer [82]. Depletion of Ciz1 reduced YAP-mediated transcription and reduced proliferation [79]. Similarly, *Ciz1* transcript and protein levels were found to be significantly elevated in gall bladder cancer (GBC) cells [80]. GBC is one of the most common and aggressive cancers of the gastrointestinal tract; however, the precise mechanism of tumor development is still unknown. Ciz1 promotes tumor growth through activation of the oncogenic Int/Wingless (Wnt)/beta-catenin T cell factor (TCF) pathway that is aberrantly activated in a range of tumors [80]. Transcriptional activation was found to be Ciz1-dependent, as overexpression of Ciz1 was shown to promote GBC growth and cell migration, whereas depletion of Ciz1 reduced proliferation, migration and tumorigenesis. It has yet to be determined whether Ciz1 contributes to transcription regulation in normal somatic cells; but in tumor cells, Ciz1 may contribute to adaptive mechanisms to enhance oncogenic signaling.

## 8. Future Perspectives

### 8.1. Deregulation of CDK Activity Promotes DNA Replication Stress

Multicellular organisms require the faithful duplication of their genome to ensure the health and viability of the organism. Failure to accurately duplicate the genome can lead to mutations that increase genetic instability through defects in DNA replication or in DNA repair pathways. In fact, intrinsic mutations that arise during DNA replication are responsible for 10%–30% of cancer incidence [83]. Further increases in intrinsic mutation rate are mediated by deregulation of the cell cycle, leading to inappropriate S-phase entry and DNA replication stress that underpins many of the early events in tumorigenesis [84]. Mutations arising during DNA replication stress are recognized by multiple pathways by activation of ataxia telangiectasia and Rad3-related protein (ATR)-signaling pathways resulting in repair [85]. Replication stress induces stalling or slowing of replication forks and consequently alters the normal cellular replication program. Replication stress is an early event in transformation of cells and contributes to genetic instability, promoting cancer development [86].

DNA replication stress is induced by deregulation of the cell cycle. This can be achieved through multiple mechanisms that increase the activity of cyclin-dependent kinases, including overexpression of cyclin subunits. For example, cyclin D1 overexpression has been shown to increase double-strand breaks in a CDK4-independent mechanism, reducing the velocity of replication forks [87]. Deregulation of the cell cycle by overexpression of cyclin E increases replication origin firing, and reduced replication fork progression due to depleted nucleotide pools [88]. Increased cyclin E protein levels promote DNA replication stress and induced recombination events in fragile sites with a low density of replication origins [89]. In addition, cyclin E overexpression induces increased transcriptional activity, leading to increased replisome and transcription factory interference [90].

Further evidence that deregulated CDK activity induces replication stress comes from studies that identified post-translational mechanisms to increase CDK activity. These include loss of inhibitor proteins expression [91], loss of inhibitory phosphorylation sites on CDK1/2 through mutation [92] or WEE1 inactivation [93], and deletion of Cdh1, which promotes accumulation of cyclin A and cyclin B [94]. Similarly, never in mitosis a related kinase 8 (NEK8) inactivation prevents down regulation of cyclin A in response to double-strand breaks, promoting genome instability [95]. Each mechanism results in premature S-phase entry, increased replication origin firing and DNA replication stress that is a direct consequence of deregulated CDK activity.

### 8.2. Does Ciz1 Contribute to Tumorigenesis by Inducing DNA Replication Stress?

Deregulation of the cell cycle has been implicated in the induction of DNA replication stress. This review has discussed the role of Ciz1 in regulation of cellular proliferation and evidence that Ciz1 directly promotes tumor growth was explored. The role of Ciz1 in cell cycle regulation through cyclin-dependent kinase interactions [42,53] suggests that Ciz1 may promote cellular proliferation and tumor growth through deregulation of the cell cycle. Ciz1 interacts with multiple regulators G1 and S phase, including p21, cyclin E, cyclin A and CDK2, influencing the localization of CDK activity [23,42,47]. Precise modulation of the cyclin-dependent kinase activity through G1 phase regulates activation of replication origins and intracellular CDK activity correlates with G1 length [96]. Use of intracellular CDK sensors in HeLa cells revealed that deregulation of CDK activity abolishes bifurcated low- and high-CDK activity levels seen in mouse fibroblasts [96,97] consistent with an increased proliferative potential for cancer cells. The increase in basal CDK activity suggests that there may be adaptive mechanisms to facilitate cell cycle progression in this proliferative signaling environment. Ciz1 contributes to the mechanisms that modulate the threshold CDK activity required for initiation of DNA replication and facilitate adaption to deregulated cyclin-CDK activity. Ciz1 can increase the range of permissive CDK concentrations that promotes initiation of DNA replication to levels that would normally block DNA synthesis—termed here as “non-permissive” high CDK activities [42,53]. Ciz1 may facilitate replicative stress as aberrant CDK activities are well established inducers of DNA replication stress.

**Figure 3:**
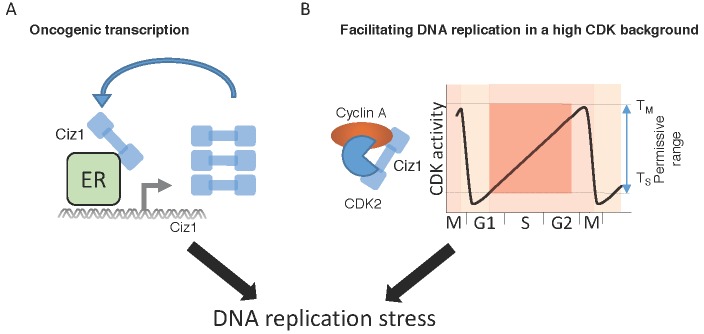
Ciz1 may contribute to DNA replication stress via stimulation of oncogenic transcription and deregulation of the cell cycle. (**A**) Oncogenic transcriptional activation mediated by Ciz1 is shown. In this example, estrogen receptor (ER) is sensitized by Ciz1 interactions leading to a positive feedback mechanism, increasing Ciz1 transcript levels. Ciz1 also increases oncogenic transcription with Yes associated protein 1/ Tafazzin (YAP/TAZ) and Beta catenin/Wnt (Int/Wingless) signaling [77,79,80]; (**B**) Ciz1 can greatly enhance the permissive concentration of cyclin A-CDK2 that can facilitate initiation of DNA replication. In this model, deregulation of cyclin expression associated with oncogenic activation increases CDK activity. Increased Ciz1 protein may enable adaption of cells to this high CDK environment and continued DNA replication at wider concentrations of cyclin-dependent kinase activity. Both mechanisms induce DNA replication stress that underpins the early events in tumorigenesis.

Ciz1 has the potential to induce DNA replication stress by two independent mechanisms (Figure 3). Ciz1 could contribute to induction of DNA replication stress by activation of oncogenic transcription [75,77,79] or by facilitating initiation of DNA replication in cells with deregulated CDK activity [42,59]. Oncogene activation increases transcriptional activity that may induce DNA replication stress [98]. For example, enhanced E2F activation can induce DNA replication stress and sustained E2F activity is required to reduce genetic instability caused during DNA replication stress [99]. Deregulation of G1 phase by oncogenic signaling through E2F pathways can also increase expression of cyclin subunits, leading to deregulation of the cell cycle, reduce G1 length and induce inappropriate S-phase entry [99].

Increased CDK activity prevents initiation of DNA replication by phosphorylation of pre-RC proteins, DNA polymerase α and Ciz1 [66,68,69,70]. As high CDK activity is non-permissive for initiation of DNA replication, cells may require adaptive mechanisms to facilitate DNA replication at inhibitory CDK concentrations. Increased Ciz1 protein levels greatly increase the permissive range for CDK activity that can promote initiation of DNA replication [42,53]. Consequently, in cells with increased oncogenic signaling and deregulated CDK activity, Ciz1 may enable S-phase entry at non-permissive CDK levels (Figure 3). Overexpression of Ciz1 in cancer cells may therefore facilitate DNA replication in a deregulated high CDK environment. This suggests that targeting Ciz1 by siRNA-mediated depletion may prevent cells from entering S-phase at non-permissive CDK levels, preventing tumor growth, consistent with results targeting Ciz1 in xenograft models [73,77,78,80,81]. This hypothesis also predicts that strategies to reduce Ciz1 levels would reduce growth in Ciz1-dependent tumors. Therefore, further research is required to elucidate the mechanisms that promote Ciz1 accumulation, which will prove a better understanding of Ciz1 function in cancer biology and identify potential targets to reduce Ciz1 levels and tumor growth.

## Figures and Tables

**Table 1:** **Ciz1 is associated with tumor growth in multiple cancers.** A summary of evidence is presented showing the effect of Ciz1 overexpression or depletion of Ciz1. Results from overexpression or depletion and consequences for tumorigenicity are presented.

Cancer	Cancer-Specific Ciz1 Alteration	Mode of Intervention	Result of Intervention	Ref.
Lung cancer	Alternative splicing	shRNA	Reduced tumor growth in xenograft models	[73]
Colorectal carcinoma (CRC)	Overexpression	siRNA	Reduced proliferation, and colony formation in vitro	[74]
Gall bladder carcinoma (GBC)	Overexpression	siRNA	Reduced xenograft tumor growth. Reduced tumor migration in vivo	[80]
Prostate cancer	Overexpression	siRNA	Reduced tumorigenesis in xenograft models, reduced proliferation, G1 checkpoint activation	[78]
Breast cancer	Overexpression	siRNA	Reduced tumorigenesis, proliferation and anchorage dependence	[76,77]
Breast cancer	Overexpression of Ciz1 increases estrogen sensitivity	Ciz1 overexpression	Increased estrogen sensitivity and increased tumor size in xenograft models.	[77]
Hepatocellular carcinoma	Overexpression	Ciz1 overexpression	Increased proliferation, migration	[79,81]
		siRNA	Reduced growth, tumorigenesis, metastasis	

shRNA: short hairpin RNA; siRNA: short interfering RNA.

## Appendix A

**Table A1:** **A list of the Ciz1 interaction partners.** This table was collated from published works and identifies human and mouse interaction partners, sourced from literature and BioGrid [100,101].

Ciz1 Binding Partner	Reference
p21	[47]
Cell division cycle 6 (Cdc6)	[23]
Cyclin E	[42]
Cyclin A	[23,42]
Cyclin-dependent kinase 2 (CDK2)	[47]
Dynein light chain	[76,102,103,104]
Estrogen receptor	[77]
Histone cluster 1	[104]
B cell Chronic lymphoid leukemia 7C	[105]
Kelch-like member 22	[105]
Scaffold attachment regulator	[105]
Mitogen activated protein kinase 14 (MAPK14)	[105]
Acid phosphatase 5, tartrate resistant (ACP5)	[105]
B lymphoma Mo-MLV insertion region 1 homolog (Bmi1)	[106]
Obscurin-like 1	[107]
Cullin 7 (Cul7)	[107]
Upf2	[108]
Enhancer of rudimentary homologue (ERH)	[109]
SH3 homology domain kinase binding protein 1	[110]
Polypyrimidine tract binding protein 1	[102]
Ectodysplasin A	[105]
SH3-domain binding protein 4	[111]

## References

[1] Bebenek K., Kunkel T.A. (2004). Functions of DNA polymerases. Adv. Protein Chem..

[2] Mehanna A., Diffley J.F. (2012). Pre-replicative complex assembly with purified proteins. Methods.

[3] Duzdevich D., Warner M.D., Ticau S., Ivica N.A., Bell S.P., Greene E.C. (2015). The dynamics of eukaryotic replication initiation: Origin specificity, licensing, and firing at the single-molecule level. Mol. Cell.

[4] Fragkos M., Ganier O., Coulombe P., Mechali M. (2015). DNA replication origin activation in space and time. Nat. Rev. Mol. Cell Biol..

[5] Heller R.C., Kang S., Lam W.M., Chen S., Chan C.S., Bell S.P. (2011). Eukaryotic origin-dependent DNA replication in vitro reveals sequential action of DDK and S-CDK kinases. Cell.

[6] Yeeles J.T., Deegan T.D., Janska A., Early A., Diffley J.F. (2015). Regulated eukaryotic DNA replication origin firing with purified proteins. Nature.

[7] Perez-Arnaiz P., Bruck I., Kaplan D.L. (2016). Mcm10 coordinates the timely assembly and activation of the replication fork helicase. Nucleic Acids Res..

[8] Onesti S., Macneill S.A. (2013). Structure and evolutionary origins of the CMG complex. Chromosoma.

[9] Sun J., Shi Y., Georgescu R.E., Yuan Z., Chait B.T., Li H., O’Donnell M.E. (2015). The architecture of a eukaryotic replisome. Nat. Struct. Mol. Biol..

[10] Sengupta S., van Deursen F., de Piccoli G., Labib K. (2013). Dpb2 Integrates the Leading-Strand DNA Polymerase into the Eukaryotic Replisome. Curr. Biol..

[11] Cvetic C., Walter J.C. (2005). Eukaryotic origins of DNA replication: Could you please be more specific?. Semin. Cell Dev. Biol..

[12] Gilbert D.M. (2004). In search of the holy replicator. Nat. Rev. Mol. Cell Biol..

[13] Cayrou C., Ballester B., Peiffer I., Fenouil R., Coulombe P., Andrau J.C., van Helden J., Mechali M. (2015). The chromatin environment shapes DNA replication origin organization and defines origin classes. Genome Res..

[14] Tatsumi Y., Ohta S., Kimura H., Tsurimoto T., Obuse C. (2003). The ORC1 cycle in human cells: I. cell cycle-regulated oscillation of human ORC1. J. Biol. Chem..

[15] Thomae A.W., Pich D., Brocher J., Spindler M.P., Berens C., Hock R., Hammerschmidt W., Schepers A. (2008). Interaction between HMGA1a and the Origin Recognition Complex Creates Site-Specific Replication Origins. Proc. Natl. Acad. Sci. USA.

[16] Edwards M.C., Tutter A.V., Cvetic C., Gilbert C.H., Prokhorova T.A., Walter J.C. (2002). Mcm2-7 Complexes Bind Chromatin in a Distributed Pattern Surrounding the Origin Recognition Complex in *Xenopus* Egg Extracts. J. Biol. Chem..

[17] Yardimci H., Walter J.C. (2014). Prereplication-complex formation: A molecular double take?. Nat. Struct. Mol. Biol..

[18] Ticau S., Friedman L.J., Ivica N.A., Gelles J., Bell S.P. (2015). Single-molecule studies of origin licensing reveal mechanisms ensuring bidirectional helicase loading. Cell.

[19] Das S.P., Borrman T., Liu V.W., Yang S.C., Bechhoefer J., Rhind N. (2015). Replication timing is regulated by the number of Mcms loaded at origins. Genome Res..

[20] Evrin C., Fernandez-Cid A., Riera A., Zech J., Clarke P., Herrera M.C., Tognetti S., Lurz R., Speck C. (2014). The ORC/Cdc6/Mcm2-7 complex facilitates Mcm2-7 dimerization during prereplicative complex formation. Nucleic Acids Res..

[21] Chang F., Riera A., Evrin C., Sun J., Li H., Speck C., Weinreich M. (2015). Cdc6 ATPase activity disengages Cdc6 from the pre-replicative complex to promote DNA replication. eLife.

[22] Deegan T.D., Yeeles J.T., Diffley J.F. (2016). Phosphopeptide binding by Sld3 links Dbf4-dependent kinase to Mcm replicative helicase activation. EMBO J..

[23] Copeland N.A., Sercombe H.E., Wilson R.H., Coverley D. (2015). Cyclin-A-CDK2-mediated phosphorylation of CIZ1 blocks replisome formation and initiation of mammalian DNA replication. J. Cell Sci..

[24] Sun J., Fernandez-Cid A., Riera A., Tognetti S., Yuan Z., Stillman B., Speck C., Li H. (2014). Structural and mechanistic insights into Mcm2-7 double-hexamer assembly and function. Genes Dev..

[25] Boos D., Sanchez-Pulido L., Rappas M., Pearl L.H., Oliver A.W., Ponting C.P., Diffley J.F. (2011). Regulation of DNA replication through Sld3-Dpb11 interaction is conserved from yeast to humans. Curr. Biol..

[26] Kumagai A., Shevchenko A., Dunphy W.G. (2010). Treslin collaborates with TopBP1 in triggering the initiation of DNA replication. Cell.

[27] Kumagai A., Shevchenko A., Dunphy W.G. (2011). Direct regulation of treslin by cyclin-dependent kinase is essential for the onset of DNA replication. J. Cell Biol..

[28] Boos D., Yekezare M., Diffley J.F. (2013). Identification of a heteromeric complex that promotes DNA replication origin firing in human cells. Science.

[29] Chowdhury A., Liu G., Kemp M., Chen X., Katrangi N., Myers S., Ghosh M., Yao J., Gao Y., Bubulya P. (2010). The DNA unwinding element binding protein DUE-B interacts with Cdc45 in preinitiation complex formation. Mol. Cell. Biol..

[30] Balestrini A., Cosentino C., Errico A., Garner E., Costanzo V. (2010). GEMC1 is a TopBP1-interacting protein required for chromosomal DNA replication. Nat. Cell Biol..

[31] Greaves E.A., Copeland N.A., Coverley D., Ainscough J.F. (2012). Cancer-associated variant expression and interaction of CIZ1 with cyclin A1 in differentiating male germ cells. J. Cell Sci..

[32] Villa F., Simon A.C., Ortiz Bazan M.A., Kilkenny M.L., Wirthensohn D., Wightman M., Matak-Vinkovic D., Pellegrini L., Labib K. (2016). Ctf4 is a Hub in the Eukaryotic Replisome that Links Multiple CIP-Box Proteins to the CMG Helicase. Mol. Cell.

[33] Pacek M., Tutter A.V., Kubota Y., Takisawa H., Walter J.C. (2006). Localization of Mcm2–7, Cdc45, and gins to the site of DNA unwinding during eukaryotic DNA replication. Mol. Cell.

[34] Takeda D.Y., Dutta A. (2005). DNA replication and progression through s phase. Oncogene.

[35] Chuang L.C., Teixeira L.K., Wohlschlegel J.A., Henze M., Yates J.R., Mendez J., Reed S.I. (2009). Phosphorylation of Mcm2 by Cdc7 promotes pre-replication complex assembly during cell-cycle re-entry. Mol. Cell.

[36] Tanaka S., Umemori T., Hirai K., Muramatsu S., Kamimura Y., Araki H. (2007). CDK-dependent phosphorylation of Sld2 and Sld3 initiates DNA replication in budding yeast. Nature.

[37] Zegerman P., Diffley J.F. (2007). Phosphorylation of Sld2 and Sld3 by cyclin-dependent kinases promotes DNA replication in budding yeast. Nature.

[38] Bruck I., Kaplan D.L. (2015). Conserved mechanism for coordinating replication fork helicase assembly with phosphorylation of the helicase. Proc. Natl. Acad. Sci. USA.

[39] Taylor M., Moore K., Murray J., Aves S.J., Price C. (2011). Mcm10 interacts with Rad4/Cut5(TopBP1) and its association with origins of DNA replication is dependent on Rad4/Cut5(TopBP1). DNA Repair.

[40] Araki H. (2010). Cyclin-dependent kinase-dependent initiation of chromosomal DNA replication. Curr. Opin. Cell Biol..

[41] Douglas M.E., Diffley J.F. (2016). Recruitment of Mcm10 to sites of replication initiation requires direct binding to the minichromosome maintenance (Mcm) complex. J. Biol. Chem..

[42] Copeland N.A., Sercombe H.E., Ainscough J.F., Coverley D. (2010). Ciz1 cooperates with cyclin-A-CDK2 to activate mammalian DNA replication in vitro. J. Cell Sci..

[43] Ellison V., Stillman B. (2001). Opening of the clamp: An intimate view of an ATP-driven biological machine. Cell.

[44] Simon A.C., Zhou J.C., Perera R.L., van Deursen F., Evrin C., Ivanova M.E., Kilkenny M.L., Renault L., Kjaer S., Matak-Vinkovic D. (2014). A Ctf4 trimer couples the CMG helicase to DNA polymerase alpha in the eukaryotic replisome. Nature.

[45] Samora C.P., Saksouk J., Goswami P., Wade B.O., Singleton M.R., Bates P.A., Lengronne A., Costa A., Uhlmann F. (2016). Ctf4 links DNA replication with Sister Chromatid Cohesion Establishment by Recruiting the Chl1 Helicase to the Replisome. Mol. Cell.

[46] Stillman B. (2015). Reconsidering DNA polymerases at the replication fork in eukaryotes. Mol. Cell.

[47] Mitsui K., Matsumoto A., Ohtsuka S., Ohtsubo M., Yoshimura A. (1999). Cloning and Characterization of a Novel p21^Cip1/Waf1^-Interacting Zinc Finger Protein, Ciz1. Biochem. Biophys. Res. Commun..

[48] Nishibe R., Watanabe W., Ueda T., Yamasaki N., Koller R., Wolff L., Honda Z., Ohtsubo M., Honda H. (2013). Ciz1, a p21Cip1/Waf1-interacting protein, functions as a tumor suppressor in vivo. FEBS Lett..

[49] Ainscough J.F., Rahman F.A., Sercombe H., Sedo A., Gerlach B., Coverley D. (2007). *C*-terminal domains deliver the DNA replication factor Ciz1 to the nuclear matrix. J. Cell Sci..

[50] Berezney R., Coffey D.S. (1975). Nuclear protein matrix: Association with newly synthesized DNA. Science.

[51] Radichev I., Parashkevova A., Anachkova B. (2005). Initiation of DNA replication at a nuclear matrix-attached chromatin fraction. J. Cell. Physiol..

[52] Munkley J., Copeland N.A., Moignard V., Knight J.R., Greaves E., Ramsbottom S.A., Pownall M.E., Southgate J., Ainscough J.F., Coverley D. (2011). Cyclin E is recruited to the nuclear matrix during differentiation, but is not recruited in cancer cells. Nucleic Acids Res..

[53] Coverley D., Marr J., Ainscough J. (2005). Ciz1 promotes mammalian DNA replication. J. Cell Sci..

[54] Takeda D.Y., Wohlschlegel J.A., Dutta A. (2001). A bipartite substrate recognition motif for cyclin-dependent kinases. J. Biol. Chem..

[55] Siddiqui K., On K.F., Diffley J.F. (2013). Regulating DNA replication in eukarya. Cold Spring Harb. Perspect. Biol..

[56] Reusswig K.U., Zimmermann F., Galanti L., Pfander B. (2016). Robust replication control is generated by temporal gaps between licensing and firing phases and depends on degradation of firing factor Sld2. Cell Rep..

[57] Diffley J.F., Cocker J.H., Dowell S.J., Rowley A. (1994). Two steps in the assembly of complexes at yeast replication origins in vivo. Cell.

[58] Mailand N., Diffley J.F. (2005). Cdks promote DNA replication origin licensing in human cells by protecting Cdc6 from APC/C-dependent proteolysis. Cell.

[59] Coverley D., Laman H., Laskey R.A. (2002). Distinct roles for cyclins e and a during DNA replication complex assembly and activation. Nat. Cell Biol..

[60] Geng Y., Lee Y.M., Welcker M., Swanger J., Zagozdzon A., Winer J.D., Roberts J.M., Kaldis P., Clurman B.E., Sicinski P. (2007). Kinase-Independent Function of Cyclin E. Mol. Cell.

[61] Coudreuse D., Nurse P. (2010). Driving the cell cycle with a minimal CDK control network. Nature.

[62] Uhlmann F., Bouchoux C., Lopez-Aviles S. (2011). A quantitative model for cyclin-dependent kinase control of the cell cycle: revisited. Philos. Trans. R. Soc. Lond. B Biol. Sci..

[63] Stern B., Nurse P. (1996). A quantitative model for the Cdc2 control of S phase and mitosis in fission yeast. Trends Genet..

[64] Wilmes G.M., Archambault V., Austin R.J., Jacobson M.D., Bell S.P., Cross F.R. (2004). Interaction of the S-phase cyclin Clb5 with an “RXL” docking sequence in the initiator protein Orc6 provides an origin-localized replication control switch. Genes Dev..

[65] Mimura S., Seki T., Tanaka S., Diffley J.F. (2004). Phosphorylation-dependent binding of mitotic cyclins to Cdc6 contributes to DNA replication control. Nature.

[66] Chen S., Bell S.P. (2011). CDK prevents Mcm2-7 helicase loading by inhibiting Cdt1 interaction with Orc6. Genes Dev..

[67] Diril M.K., Ratnacaram C.K., Padmakumar V.C., Du T., Wasser M., Coppola V., Tessarollo L., Kaldis P. (2012). Cyclin-dependent kinase 1 (Cdk1) is essential for cell division and suppression of DNA re-replication but not for liver regeneration. Proc. Natl. Acad. Sci. USA.

[68] Walter D., Hoffmann S., Komseli E.S., Rappsilber J., Gorgoulis V., Sorensen C.S. (2016). SCF(Cyclin F)-dependent degradation of CDC6 suppresses DNA re-replication. Nat. Commun..

[69] Johansson P., Jeffery J., Al-Ejeh F., Schulz R.B., Callen D.F., Kumar R., Khanna K.K. (2014). SCF-FBXO31 E3 Ligase Targets DNA Replication Factor Cdt1 for Proteolysis in the G_2_ Phase of Cell Cycle to Prevent Re-replication. J. Biol. Chem..

[70] Drury L.S., Perkins G., Diffley J.F. (2000). The cyclin-dependent kinase Cdc28p regulates distinct modes of Cdc6P proteolysis during the budding yeast cell cycle. Curr. Biol..

[71] Voitenleitner C., Fanning E., Nasheuer H.P. (1997). Phosphorylation of DNA polymerase alpha-primase by cyclin A-dependent kinases regulates initiation of DNA replication in vitro. Oncogene.

[72] Voitenleitner C., Rehfuess C., Hilmes M., O’Rear L., Liao P.C., Gage D.A., Ott R., Nasheuer H.P., Fanning E. (1999). Cell cycle-dependent regulation of human DNA polymerase alpha-primase activity by phosphorylation. Mol. Cell. Biol..

[73] Higgins G., Roper K.M., Watson I.J., Blackhall F.H., Rom W.N., Pass H.I., Ainscough J.F., Coverley D. (2012). Variant Ciz1 is a circulating biomarker for early-stage lung cancer. Proc. Natl. Acad. Sci. USA.

[74] Yin J., Wang C., Tang X., Sun H., Shao Q., Yang X., Qu X. (2013). CIZ1 regulates the proliferation, cycle distribution and colony formation of RKO human colorectal cancer cells. Mol. Med. Rep..

[75] Wang D.Q., Wang K., Yan D.W., Liu J., Wang B., Li M.X., Wang X.W., Liu J., Peng Z.H., Li G.X. (2014). Ciz1 is a novel predictor of survival in human colon cancer. Exp. Biol. Med..

[76] Den Hollander P., Kumar R. (2006). Dynein light chain 1 contributes to cell cycle progression by increasing cyclin-dependent kinase 2 activity in estrogen-stimulated cells. Cancer Res..

[77] Den Hollander P., Rayala S.K., Coverley D., Kumar R. (2006). Ciz1, a novel DNA-binding coactivator of the estrogen receptor alpha, confers hypersensitivity to estrogen action. Cancer Res..

[78] Liu T., Ren X., Li L., Yin L., Liang K., Yu H., Ren H., Zhou W., Jing H., Kong C. (2015). Ciz1 promotes tumorigenicity of prostate carcinoma cells. Front. Biosci..

[79] Lei L., Wu J., Gu D., Liu H., Wang S. (2016). Ciz1 interacts with yap and activates its transcriptional activity in hepatocellular carcinoma cells. Tumour Biol..

[80] Zhang D., Wang Y., Dai Y., Wang J., Suo T., Pan H., Liu H., Shen S., Liu H. (2015). Ciz1 promoted the growth and migration of gallbladder cancer cells. Tumour Biol..

[81] Wu J., Lei L., Gu D., Liu H., Wang S. (2015). Ciz1 is upregulated in hepatocellular carcinoma and promotes the growth and migration of the cancer cells. Tumour Biol..

[82] Pobbati A.V., Hong W. (2013). Emerging roles of TEAD transcription factors and its coactivators in cancers. Cancer Biol. Ther..

[83] Wu S., Powers S., Zhu W., Hannun Y.A. (2016). Substantial contribution of extrinsic risk factors to cancer development. Nature.

[84] Burrell R.A., McClelland S.E., Endesfelder D., Groth P., Weller M.C., Shaikh N., Domingo E., Kanu N., Dewhurst S.M., Gronroos E. (2013). Replication stress links structural and numerical cancer chromosomal instability. Nature.

[85] Zeman M.K., Cimprich K.A. (2014). Causes and consequences of replication stress. Nat. Cell Biol..

[86] Macheret M., Halazonetis T.D. (2015). DNA replication stress as a hallmark of cancer. Annu. Rev. Pathol..

[87] Shimura T., Ochiai Y., Noma N., Oikawa T., Sano Y., Fukumoto M. (2013). Cyclin D1 overexpression perturbs DNA replication and induces replication-associated DNA double-strand breaks in acquired radioresistant cells. Cell Cycle.

[88] Bester A.C., Roniger M., Oren Y.S., Im M.M., Sarni D., Chaoat M., Bensimon A., Zamir G., Shewach D.S., Kerem B. (2011). Nucleotide deficiency promotes genomic instability in early stages of cancer development. Cell.

[89] Teixeira L.K., Wang X., Li Y., Ekholm-Reed S., Wu X., Wang P., Reed S.I. (2015). Cyclin E deregulation promotes loss of specific genomic regions. Curr. Biol..

[90] Jones R.M., Mortusewicz O., Afzal I., Lorvellec M., Garcia P., Helleday T., Petermann E. (2013). Increased replication initiation and conflicts with transcription underlie cyclin E-induced replication stress. Oncogene.

[91] Quereda V., Porlan E., Canamero M., Dubus P., Malumbres M. (2016). An essential role for Ink4 and Cip/Kip cell-cycle inhibitors in preventing replicative stress. Cell Death Differ..

[92] Hughes B.T., Sidorova J., Swanger J., Monnat R.J., Clurman B.E. (2013). Essential role for Cdk2 inhibitory phosphorylation during replication stress revealed by a human Cdk2 knockin mutation. Proc. Natl. Acad. Sci. USA.

[93] Beck H., Nahse-Kumpf V., Larsen M.S., O’Hanlon K.A., Patzke S., Holmberg C., Mejlvang J., Groth A., Nielsen O., Syljuasen R.G. (2012). Cyclin-dependent kinase suppression by WEE1 kinase protects the genome through control of replication initiation and nucleotide consumption. Mol. Cell. Biol..

[94] Greil C., Krohs J., Schnerch D., Follo M., Felthaus J., Engelhardt M., Wasch R. (2016). The role of APC/C(Cdh1) in replication stress and origin of genomic instability. Oncogene.

[95] Choi H.J., Lin J.R., Vannier J.B., Slaats G.G., Kile A.C., Paulsen R.D., Manning D.K., Beier D.R., Giles R.H., Boulton S.J. (2013). NEK8 links the ATR-regulated replication stress response and S phase CDK activity to renal ciliopathies. Mol. Cell.

[96] Spencer S.L., Cappell S.D., Tsai F.C., Overton K.W., Wang C.L., Meyer T. (2013). The proliferation-quiescence decision is controlled by a bifurcation in CDK2 activity at mitotic exit. Cell.

[97] Barr A.R., Heldt F.S., Zhang T., Bakal C., Novak B. (2016). A dynamical framework for the all-or-none G1/S transition. Cell Syst..

[98] Kotsantis P., Silva L.M., Irmscher S., Jones R.M., Folkes L., Gromak N., Petermann E. (2016). Increased global transcription activity as a mechanism of replication stress in cancer. Nat. Commun..

[99] Bertoli C., Herlihy A.E., Pennycook B.R., Kriston-Vizi J., de Bruin R.A. (2016). Sustained E2F-dependent transcription is a key mechanism to prevent replication-stress-induced DNA damage. Cell Rep..

[100] Chatr-Aryamontri A., Oughtred R., Boucher L., Rust J., Chang C., Kolas N.K., O’Donnell L., Oster S., Theesfeld C., Sellam A. (2016). The BioGRID interaction database: 2017 Update. Nucleic Acids Res..

[101] Chatr-Aryamontri A., Breitkreutz B.J., Oughtred R., Boucher L., Heinicke S., Chen D., Stark C., Breitkreutz A., Kolas N., O’Donnell L. (2015). The BioGRID interaction database: 2015 Update. Nucleic Acids Res..

[102] Hein M.Y., Hubner N.C., Poser I., Cox J., Nagaraj N., Toyoda Y., Gak I.A., Weisswange I., Mansfeld J., Buchholz F. (2015). A human interactome in three quantitative dimensions organized by stoichiometries and abundances. Cell.

[103] Gupta G.D., Coyaud E., Goncalves J., Mojarad B.A., Liu Y., Wu Q., Gheiratmand L., Comartin D., Tkach J.M., Cheung S.W. (2015). A dynamic protein interaction landscape of the human centrosome-cilium interface. Cell.

[104] Lambert J.P., Tucholska M., Go C., Knight J.D., Gingras A.C. (2015). Proximity biotinylation and affinity purification are complementary approaches for the interactome mapping of chromatin-associated protein complexes. J. Proteom..

[105] Huttlin E.L., Ting L., Bruckner R.J., Gebreab F., Gygi M.P., Szpyt J., Tam S., Zarraga G., Colby G., Baltier K. (2015). The bioplex network: A systematic exploration of the human interactome. Cell.

[106] Cao Q., Wang X., Zhao M., Yang R., Malik R., Qiao Y., Poliakov A., Yocum A.K., Li Y., Chen W. (2014). The central role of EED in the orchestration of polycomb group complexes. Nat. Commun..

[107] Hanson D., Stevens A., Murray P.G., Black G.C., Clayton P.E. (2014). Identifying biological pathways that underlie primordial short stature using network analysis. J. Mol. Endocrinol..

[108] Lehner B., Sanderson C.M. (2004). A protein interaction framework for human mRNA degradation. Genome Res..

[109] Krzyzanowski M.K., Kozlowska E., Kozlowski P. (2012). Identification and Functional Analysis of the *erh1*^+^ Gene Encoding Enhancer of Rudimentary Homolog from the Fission Yeast *Schizosaccharomyces pombe*. PLoS ONE.

[110] Havrylov S., Rzhepetskyy Y., Malinowska A., Drobot L., Redowicz M.J. (2009). Proteins recruited by SH3 domains of Ruk/CIN85 adaptor identified by LC-MS/MS. Proteome Sci..

[111] Thalappilly S., Suliman M., Gayet O., Soubeyran P., Hermant A., Lecine P., Iovanna J.L., Dusetti N.J. (2008). Identification of multi-SH3 domain-containing protein interactome in pancreatic cancer: A yeast two-hybrid approach. Proteomics.

